# Paradigm shift in head and neck oncology patient management

**DOI:** 10.1186/s40463-017-0229-8

**Published:** 2017-09-19

**Authors:** Chiquit van Linden van den Heuvell, Florence van Zuuren, Mary Wells, Geert van der Laan, Harry Reintsema

**Affiliations:** 1 Department Oral & Maxillofacial Surgery and Special Dental Care, University Medical Center Groningen, P.O. box 30.001, 9700 RB Groningen, The Netherlands; 2 Faculty of Social and Behavioral Sciences, University of Amsterdam, Weesperplein 4, 1018 XA Amsterdam, The Netherlands; 3 School of Nursing and Midwifery, 11 Airlie Place, Dundee, UK; 4 Present Address: NMAHP Research Unit, University of Stirling, Stirling, FK9 4LA UK

**Keywords:** Shared decision making, Head and neck oncology, Patient involvement

## Abstract

**Objective:**

This article describes a paradigm shift in what is considered to be good care for patients living with and after (head and neck) cancer. HNO patients often experience severe and difficult physical and psychosocial problems due to the nature and location of the disease. Many disciplines are involved in their treatment, so their voice is only one amongst many others in the decision making process. For this patient group it seems complicated to put the concept of Shared Decision Making into practice. As a step in this direction, patient reported outcomes which ask patients to select the disconcerting issues and symptoms can be used as a basis for referral, supportive care and treatment decision making. We need to provide more tailored and personalized information that is specific to individual circumstances, preferences and concerns and focuses more on the impact of treatment and access to help and support. Follow up of these patients should be concentrated on both medical and emotional aspects.

**Practice implications:**

A shift in the way caregivers provide their information contributes to a more profound involvement of patients in treatment decisions.

## Background

The relevance of patient involvement in treatment decisions is becoming a more important issue [1, 2]. “The best interest of the patient is *the* single most important factor in making treatment decision” seems to be a straightforward principal underlying this discussion.

In our opinion it remains unclear what this principal means for treatment team and the individual patient in everyday practice.

In this article we want to describe how the principal of integrating the patient’s perspective can be given tangible form by giving the missing team member a voice, reflecting on what type of counselling can improve patients’ treatment decisions.

## Main topic

### Giving the missing team member a voice

Involving patients with head and neck cancer in decisions throughout the treatment pathway can be particularly problematic.

Firstly, as a group, these patients have tended to be seen as socially disadvantaged and vulnerable, with poor health behaviors, including heavy drinking and smoking. Arguably, this has encouraged a paternalistic attitude amongst health care professionals, that patients with head and neck cancer are likely to be passive and even non-compliant in their behaviors. Additionally, public awareness of head and neck cancer is poor [3]. Even studies of medical and dental students reveal a low level of awareness [4, 5]. And stories of celebrities with head and neck cancer seem to have less impact on the public than stories of celebrities with other better-known cancers [6]. In all, patients with head and neck cancer tend not to be seen as a vocal, proactive or influential group. Research has shown that they have high psychological morbidity [7], high rates of suicide [8] and are likely to delay seeking treatment [9].

Secondly, as individuals, people with head and neck cancer often have to deal with particularly severe and difficult physical and psychosocial problems [10]. Numerous qualitative studies have shown that patients with head and neck cancer experience a loss of their sense of ‘self’ [11–13]. The contribution of physical and functional changes to this sense of loss is significant, including weight loss, changes in appearance or speech, and difficulties with social activities such as eating and drinking and kissing, but many patients also describe losing their sense of who they are as a person. The experience of cancer can be lonely and uncertain, and the impact of HNC may be particularly distressing, because social interaction is so often impaired. Patients describe their energies being so taken up with getting through the day, or coping with treatment and its effects, that they place less emphasis on wider needs and concerns. In addition, many put on a brave face, compare themselves to others who they perceive as worse off, or fail to legitimize their symptoms because in comparison to the cancer, they seem trivial [11].

Creating an environment in which symptoms are legitimized, individuals’ concerns are assessed and peoples’ selves are recognized, is not an easy task within a busy head and neck cancer clinic. In order to provide the complex multidisciplinary care that most patients with head and neck cancer require, a large number of different health professionals are likely to be involved, and this may make it difficult for patients’ own voices to emerge amongst the many perspectives being shared. Additionally, the context of the clinic consultation is not always conducive to an open exchange between the clinician and the patient. An interview- and observational study of clinic consultations, including those in an ear, nose and throat clinic, showed that both patients and health professionals often fail to disclose information which is highly relevant to decision making [14]. Information related to the patient’s problem e.g. symptoms, beliefs and concerns, and/or information about treatment options e.g. how treatments actually work, uncertainties about effectiveness, was often voiced by patients and professionals before and after consultations, but not exchanged *during* the consultation. There were a number of reasons for this non-disclosure. These included the environment of the consultation e.g. too many people within the room; the attitudes or beliefs of health care professionals; the wish to portray a particular image or achieve a particular outcome e.g. by not revealing a symptom the patient might be given a longer time period between follow-up appointments; and a belief that certain information was not worth mentioning or was felt to be inappropriate to the discussion [14]. Numerous studies also show that clinicians are poor at recognizing emotional distress and tend to underestimate symptoms, particularly if they are not directly observable [15, 16]. Shared decision making is unlikely to occur when there is no two-way exchange of relevant information.

We therefore need to find better ways of eliciting patients’ views, experiences, priorities and concerns. Attitudes to potential treatment outcomes vary between individuals and it is vital that we do not make assumptions about the preferences and priorities of different people facing the same diagnosis and treatment. If we are to ensure that patients’ voices and experiences are more effectively integrated into the treatment pathway, we need to make routine use of patient reported outcomes, such as the Patient Concerns Inventory [17] which asks patients to select the issues and symptoms that are causing them concern, and can therefore be used as a basis for referral, supportive care and treatment decision making [18, 19]. Additionally, we need to re-consider the type of information we offer, providing more tailored and personalized information that is specific to individual circumstances, preferences and concerns and focuses more on the impact of treatment and access to help and support [20].

Finally, the way we currently follow up patients with head and neck cancer should be reviewed. Models of follow-up care vary widely across the world and no randomized trials exist to guide current practice [21]. Surveillance and monitoring of recurrence clearly remains extremely important, but supportive care and rehabilitation is just as crucial if people are to live with and beyond cancer. In the UK, the National Cancer Survivorship Initiative was launched in 2010 and has identified five key shifts that are required in the approach to care for people living with cancer [22] (see Table 1).

**Table 1:** Five Key Shifts in the approach to care for people living with and beyond cancer

• A cultural shift in the approach to care and support for people affected by cancer to a greater focus on recovery, health and well-being after cancer treatment
• A shift towards assessment, information & personalised care planning
• A shift towards support for self-management, from a clinically led approach to follow-up care to supported self-management, based on individual needs and preferences and with the appropriate clinical assessment, support and treatment
• A shift from a single model of clinical follow up to tailored support that enables early recognition of and preparation for the consequences of treatment as well as early recognition of signs and symptoms of further disease
• A shift from an emphasis on measuring clinical activity to a new emphasis on measuring experience and outcomes for cancer survivors through routine use of patient reported outcome measures in after care services

**Fig. 1:**
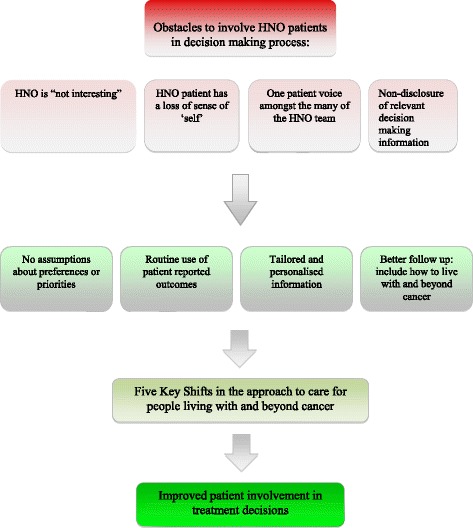
Improving patient involvement in treatment decisions. Graphical representation of obstacles encountered in implementing the patients’ voice into the decision making process, and suggested steps to improve patient involvement in treatment decisions

Implementing these shifts will require us to listen much more carefully to the voices and experiences of the patients in our care. It will also require us as health care professionals to work more effectively together across disciplines and across care settings, so that we can start to make patient involvement in decisions about treatment and care more of a reality (see Fig. 1).

“The more the group is willing to accept the different voices emerging from the multidisciplinary clinic, the more it will be able to listen to the patient’s voice” [23].

### Improving treatment decisions in communication with the patient

Ideally, a decision to undergo a certain type of treatment should reflect the patients’ preference with full knowledge of the impact and outcome of all alternatives. In reality, you can only choose and undergo one alternative at a time, after which you are no longer the same person as before this treatment. The individual patient therefore is limited to decide on the basis of prognostic information on the group level, provided by health care professionals.

**Fig. 2:**
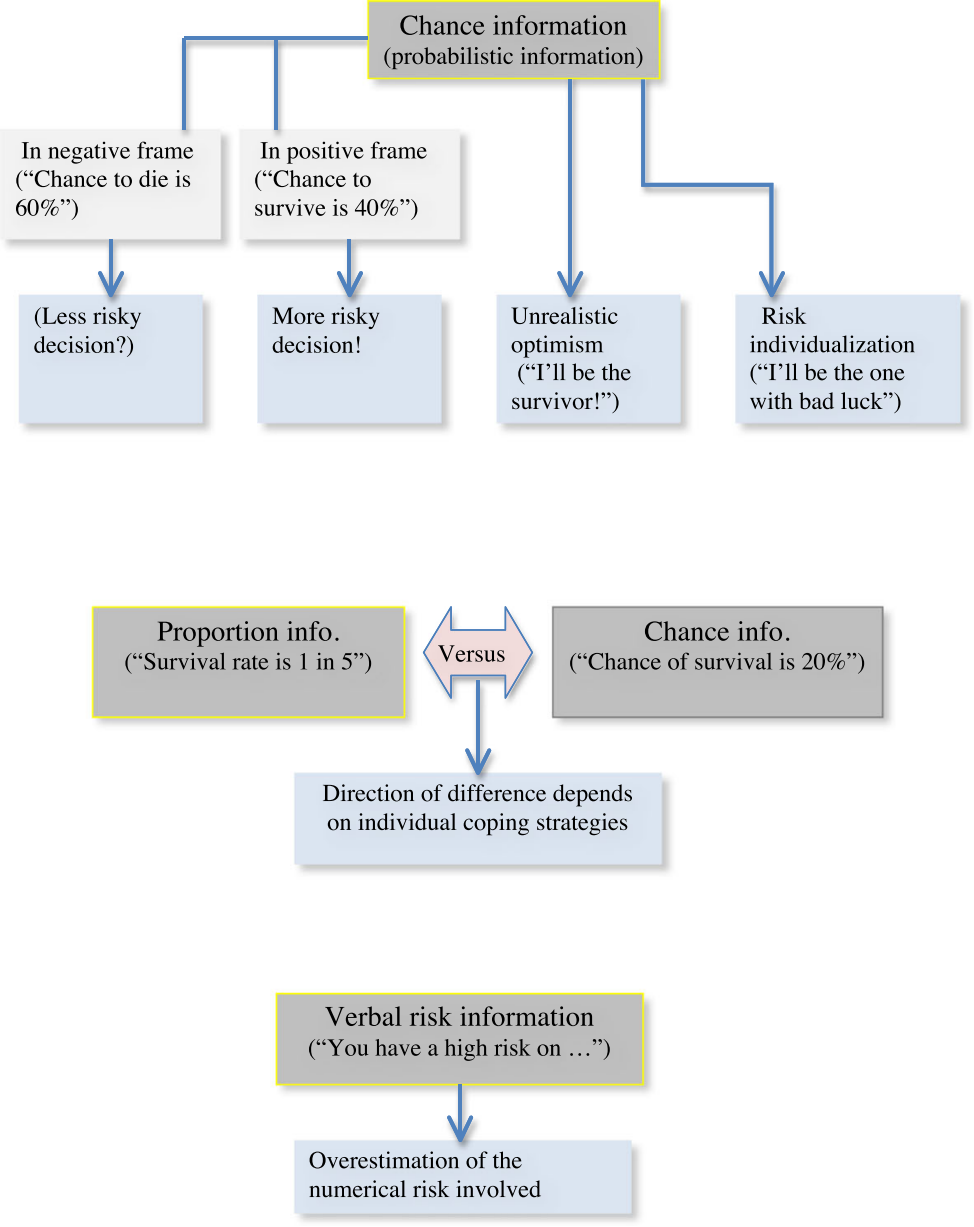
Consequences of wording. Schematic overview illustrating consequences of ways professionals use to provide information to patients

A factor of vital importance in this respect is the way professionals provide the information (see Fig. 2). It has been shown in the field of clinical genetics that counselors may be non-directive in the sense of abstaining from steering advice, but that nevertheless they exert procedural directivity in the sense of stimulating the patient to make further use of the health care system – instead of abandoning it [24]. Often, patients receive no information on alternative treatments, among these the option of abstaining from (further) treatment altogether. Professionals may even lack the knowledge about the ‘natural’ course of the illness.

The same mechanism may be at work in head and neck oncology and may partly explain why so many patients choose for invasive treatments, in spite of all the hardships and bad outcomes.

Patients’ dependency on the way the information is provided increases with chance information, such as survival rates after surgery or risk information on side effects. Patients they have their own chance interpretations, which may range from unrealistic optimism [25, 26] to risk-individualization, i.e. the belief that one will be the very one person with the bad outcome.

The framing of chance information is another factor of influence. A positive frame (‘*This treatment gives you a chance of survival of 40%* ‘) leads to a more risk taking decision than a negative one (‘… *the chance that you’ll die will be 60%*’ [27, 28]. Information in terms of chances (‘*…your survival rate is 20%*’) is judged differently from information in terms of proportions (‘… *your survival rate is 1 in 5’*), but the direction of this difference depends on individual cognitive strategies [29]. The use of verbal risk information (‘*You have a high risk on…*’) generally leads to an overestimation by the patient of the numerical risk involved [30].

Within the context of these communication aspects, patients tend to respond in an active way. Treatment may be seen as a last straw, as a form of reassurance even, because it offers support, regular contact with the health care system and a plan of action. In contrast, the alternative of abstaining from treatment may induce uncertainty because one feels left to ones fate. The negative sides of invasive treatment are discovered when it is already too late. Once treatment has been started, it may be very difficult to decide to stop it. In the fields of clinical genetics and neonatology it has been observed that for (parents of) patients as well as for professionals there is a bias to continue an already started treatment instead of terminating it, even in the light of the medical futility of this treatment [24, 31, 32]. Terminating treatment would feel the same as giving up all hope, and hope is the driving force in life [33].

Of all HNO patients every second person will be in dire need for palliative care within five years [34], without a clear prognostic indicator being available for the individual patient. It could be argued therefore that curative and palliative pathways should be integrated from diagnosis on, providing good palliation when needed, without sacrifice any chances for cure.

The integration of palliative care in treatment planning would mean an enhancement of the choices patients have in the decision making process. Since the cancer illness curve is characterized by a sharp decline towards the end, early integration of palliative aspects in an integrated care plan could prevent many harmful interventions from the curative options towards the inevitable end, whereas, in the earlier phase, no concessions should be made to curative chances.

It may be concluded that, in communicating treatment decisions with patients, health care professionals should be aware of the above mentioned factors. They should be realistic about side effects of treatment and treatment outcome, and provide chance information in different frames. Also, they should be open to the option of abstaining from treatment or terminating an already started treatment and help patients to handle this new, undefined situation.

### Patient’s perspective

In order to involve patients’ preference in treatment decisions, health care professionals not only should be aware of how they provide information - as outlined above -, they also may need actively exploring patients’ motives regarding (the result of) treatment. For both patient and health care professional it may be hard to estimate the goals and motives of ‘the opposite party’, making it harder to achieve clarity of information. The following case illustrates the consequences of an implicit misunderstanding.

### Case

Mister O, diabetic since his twenties, was referred with a tumour in his nose. The tumour had to be removed surgically with subsequent radiotherapy. He cooperated in an interview with a clinical psychologist (CLH) after therapy to discuss the issue of: “Has it been worth it?”.

The interview took place a few months after resection of his nose, the frontal two third of the maxilla and the major part of his upper lip. It appeared that mister O could live very well without his nose: “My face is not who I am”. But what hampered him most was his discovery of the maxilla missing after the operation: “Nobody had told me a thing about that, it’s a shame!”. His denture was difficult to retain by his upper lip that was now a small band of tissue without practical function.

Mister O showed himself determined about the suggestion to reconstruct his maxilla and upper lip in one time: “Over my dead body”. He considered a lengthy operation like that too much of a risk in his situation: “I don’t care so much for that upper lip”. He only considered a reconstruction of the lip if that would mean an enhanced support of the prosthesis.

In order to better fixate the prosthesis installation of zygomatic implants was suggested, which he also refused: he expected a higher than average failure risk of the implants, due to the radiation therapy.

One year later mister O died from a complication of the same operation he had refused decisively during the interview: an extensive reconstructive surgery, aiming at restoring both the bony structure of the maxilla as well as the upper lip, by means of a free vascularised fibula flap.

Another year later his widow was interviewed about the background of the operation. She was eager to explain the motives of her husband: “Due to his diabetes he suffered from erectile dysfunction. I didn’t mind, but he did. After the nose resection he could no longer use his upper lip either: he couldn’t kiss me anymore and that he found awful: ‘If I even can’t do *that* anymore…’. And that’s why he decided for the operation: so he could kiss me again”.

Had the treatment team been aware of mister O’s motive to agreeing with the operation, his expectations could have been nuanced. Restoration of the upper lip would have been mainly aesthetic and functional in the sense that it would lend more support to the prosthesis and a better lip closure. But the specific sensory in- and output desired would have been rather questionable, to say the least.

## Conclusion

The concept of patient involvement in treatment decisions is widely acknowledged. Less attention is paid to how to realize this. In Head & Neck Oncology treatment some factors impede shared decision making. Many disciplines are involved, making the patient’s voice only one amongst many. During clinic consultations both health care professionals and patient seem to fail to disclose information that is highly relevant to decision making. This is all the more important as recent literature suggests an association between depression, post operative functional performance status and even survival in HNO patients [35, 36]. The etiology and strength of this relationship is not yet clear. It would be relevant to know if the usual unilateral nature of the decision making process enhances the level of depression in HNO patients, augmenting a perceived lack of influence and control.

A paradigm shift seems necessary for the transition from traditional decision making to shared decision making. A change should take place in what is considered to be appropriate care for patients living with and beyond cancer, such as a broadening of the focus on merely the disease to recovery and well-being after cancer treatment, a shift to patient’s needs and preferences for self-management being decisive for follow up care and creating follow up support that enables patients to early recognize consequences of treatment and signs of further disease.

To realize the shift to shared decision making, the oncology treatment team needs to listen much more carefully to the voices and experiences of the patients in care, the team should be aware of the influence it exerts the way information is provided, and the individual members need to work more effectively together across disciplines and across care settings.
